# Multi-Perspective Characterization of a Performance of a Barrel Drill Made of Tungsten Carbide Composite

**DOI:** 10.3390/ma18040794

**Published:** 2025-02-11

**Authors:** Kazimierz Rychlik, Mirosław Bramowicz, Sławomir Kulesza

**Affiliations:** 1 Department of Building Elements Engineering, Building Research Institute, 1 Filtrowa Street, 00-611 Warsaw, Poland; k.rychlik@itb.pl; 2 Faculty of Technical Sciences, University of Warmia and Mazury in Olsztyn, 11 Oczapowskiego Street, 10-719 Olsztyn, Poland; miroslaw.bramowicz@uwm.edu.pl

**Keywords:** sintered tungsten carbide, gun barrel drill, multiscale characterization

## Abstract

This paper presents the results of an estimation of the structural and roughness parameters of the outer surface layers of a barrel drill made of cobalt matrix sintered tungsten carbide samples (WC-Co) made by sintering and subjected to finishing by grinding. In order to evaluate the geometric and functional structure of the surface, profilometric measurements were carried out at different scan lengths. The geometric structure of the studied surfaces was characterized by the roughness parameters Ra, Rq, and Rz, while the functional structure was determined by the reduced profile heights Rpk, Rk, Rvk and the material ratios M_r1_ and M_r2_ determined by the Abbott-Firestone curves. Multiscale analysis of the dependence of the roughness and functional parameters on the measurement lengths was carried out using the root mean square (RMS) method, from which monofractal structures of the surface profile variations were found. Consistency of the fractal dimensions estimated for the drill bit might be due to its finer finishing.

## 1. Introduction

Among tool materials, metal carbides are a first-choice option in many applications including, for example, machining and cutting tools. In the early 1990s, half of the cutting tools were made from various carbides offering beneficial characteristics such as high strength, good thermal conductivity and low thermal expansion, excellent cutting properties, versatility, and relatively low manufacturing costs [1]. Among them, special attention was paid to the carbides of refractory metals, such as tungsten (W), titanium (Ti), tantalum (Ta), and niobium (Nb), with a volume fraction between 65 and 98 per cent, in which cobalt (Co), nickel (Ni), molybdenum (Mo), iron (Fe) and their alloys with cobalt served as binders (matrix). The properties of cemented carbides depend on their chemical/phase compositions and the shape/size of the grains [2]. The bending strength of cemented carbides increases with the increasing amount of Co and Ta in their structure and decreasing fraction of Ti [3], but it is also affected by the grain size of the matrix. Another advantage of the carbides is high compressive strength; however, with the increasing content of Co and Ti, these solids become brittle. On the other hand, sintered carbides show high abrasion resistance when small amounts of Co atoms are added to the structure [4].

Manufacturers of machining tools offer many different types of carbides, labelled with their own schemes, which makes unambiguous identification of the materials virtually possible. For example, the PN-ISO 513:1999 standard brings the classification of cemented carbides, which introduces three groups of applications completely insufficient in the light of current requirements [5]. On the other hand, the PN-EN 88/H-89500 standard defines 17 grades of carbides for machining [6]. Global tool manufacturers extend these schemes to introduce new application groups and additional carbide grades. Hence, an identification of the tool materials brought by different suppliers becomes a vital issue. This includes verification of catalogue data provided by the manufacturers, which raises several difficulties because it requires a large volume of the sample, inhomogeneous properties across the sample, has a complex shaped tool, destructive measurement procedures, etc. Examples of such tools are gun barrel drills popular in deep-hole drilling technology, whose specific shape and unique characteristics (a tool that behaves more like the cutter than a drill) make any testing procedures challenging.

In drilling technology, the surface roughness of the drill bit is found to be one of the key output response parameters affecting product quality, esthetics, and overall figure-of-merit of the tool [7]. Moreover, the surface of the drilled holes has an influence on the performance of the component product and also determines the life cycle of the manufactured product [7]. In drilling technology research, many experimental studies have been conducted to analyze the surface quality of the tools and formed parts, including neural networks [8], Taguchi methodology and statistical analysis of variance [9], digital image processing algorithms [10], vibration signal analysis [8], and others. However, in the field of deep drilling, a comprehensive review article is scarcely available to help researchers in their studies. So far, reported studies have taught us that the drilling process affects the surface of the tool and the machined workpieces such that

- Surface roughness is highly correlated with a tool wear data [11],
- The drill type, followed by drill diameter, shows a higher percentage contribution to surface roughness [12],
- The internal friction angle and cohesive strength of cement-rock composites both increase with the rise in surface roughness [13],
- Failure to control the flank wear occurring during the drilling process causes both the cutting tool and the workpiece to be significantly affected [14].

The geometric structure of the residual layer of a material is partly due to the way it was formed, but it is also affected by the wear processes occurring between surfaces in contact during its further processing. Specific surface shapes at various wavelengths and sampling rates might be analyzed in terms of statistical parameters such as arithmetic average roughness (Ra), root-mean-square roughness (Rq), maximum peak-to-valley height (Rt) average peak-to-valley distance (Rz) etc., essentially derived from the distribution function of the height samples from profilometric measurements [15,16,17].

For the sake of simplicity, most researchers use arithmetic mean roughness for experimental evaluation of the surface variability. In engineering practice, however, these parameters are usually specified without guidance on the sampling length, making inambiguous comparisons of the obtained results highly difficult. Also, there are concerns about clear-cut correspondence between roughness parameters and the surface geometry of the detail under study. It turns out that different shapes with non-Gaussian profiles can be described by similar roughness parameters. To overcome this problem, an alternative roughness characterization was proposed that makes use of the bearing area curve BAC (or Abbott-Firestone curve) [18]. These parameters provide functional characteristics of the surface including its load-carrying capacity, ability to store lubricants, and wear during the lapping phase. A major benefit of this method lies in its computational simplicity and functional accuracy in revealing topography features important in many tribological problems (wear, friction, lubrication, etc.), however, it also suffers from being scale-dependent. The last option is brought by fractal analysis [19] that shines light onto similarities between repetitive patterns of roughness, waviness, and habit observed at different scale-lengths. This method produces insensitivity in the way the surface is measured (sampling area, resolution, etc.); however, the final results strongly depend on the numerical procedure of the following data processing [20].

The geometric and functional structures of the surface of the cemented carbides are the fingerprints of the state of the residual surface layer. Although fractal analysis with the help of the RMS method has been used so far in many studies of processed workpieces made of engineered materials, e.g., porous materials [21], iron films [22], Ni-Mn-Ga type Heusler alloys [23] etc., little can be found about its application in ex situ profilometric measurements of real cutting tools. The fractal method was considered an interesting option for widely used geometric and functional analyses of surfaces, e.g., for rapid hardware testing or prototyping. It allows us to distinguish between scale-invariant patterns in topography data and those that change with the sample size, exhibiting the dependence of the roughness and the bearing parameters on the length of the unit section.

One of the critical applications of metrology in tooling is the dimensional characterization of cutting tools to ensure they perform optimally and have a long service life. Roughness measurements can also be used to predict how removed material will exit the tool in the aim of preventing clot formation or overheating. The presented work aims at determining the diverse structural and surface shape measurements of the cutting blade of the gun barrel drill made of the sintered WC-Co composite that includes, among others, hardness measurements (Vickers method), structural and chemical analysis (XRD method), and surface roughness parameters.

## 2. Materials and Methods

### 2.1. Gun Barrel Drill

**Figure 1:**
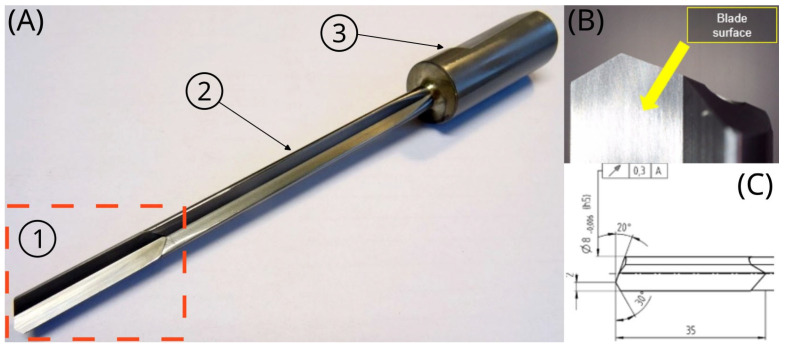
Images of the EB80 monolithic barrel drill bit: (**A**) general view (1—drill tip, 2—adapter, 3—notch shank), (**B**) the tip of the drill, (**C**) technical drawing of the drill blade.

An investigation was carried out to determine structural and topographical properties of the cutting edge of a monolithic gun barrel drill (EB80 manufactured by GÜHRING, Albstadt, Germany) made of a cobalt-matrix tungsten carbide composite (WC-Co) labelled as K15 in accordance with the PN-ISO 513:1999 standard. Figure 1 shows various views of the drill with the blades encircled in a frame. The geometric specification of the barrel drill includes

- A single blade (8 mm in diameter) with two asymmetric cutting edges,
- An uncoated cutting edge made of monolithic sintered WC-Co composite,
- A G-type guide strips with 1:800 alignment,
- A whistle notch shank.

The declared specification of the base material provided by the manufacturer is presented in Table 1.

### 2.2. Metallographic Imaging

A metallographic examination of the material was performed providing that the surface layer was removed using Murakami’s reagent (100 mL H_2_O, 10 g NaOH, 10 g K_3_[Fe(CN)_6_]). The uncovered structure was recorded and the obtained image was then analyzed using ImageJ software (V. 1.53) in order to determine the granularity of the sintered carbides under investigation.

### 2.3. Hardness Measurements

Surface hardness measurements were carried out to comply with the following standards: [6] and [7] using the Vickers method and INNOVATEST 400 tester (Veldhoven, The Netherlands). The following loads were used: 980.7 mN (HV0.1), 4.903 N (HV0.5), and 294.2 N (HV30) at seven independent locations, from which the average hardness was determined according to

(1) $$HV=0.1891\cdot \frac{F}{d_{av}^{2}}$$

where F—loading force in [N] and d_av_—arithmetic mean of the two diagonal imprints in [mm].

### 2.4. X-Ray Diffraction Measurements

XRD (X-Ray Diffraction) measurements were carried out using a Panalytical X’Pert Pro powder diffractometer (Almelo, The Netherlands) with a Cu anode tube but without the Cu-Kα2 filter. For this reason, the weighted average wavelength was used in the calculations 1.541874 Å [24]. The measurements were performed in the θ–2θ configuration covering the 2θ range from 7 to 120° with 0.016° steps and integration intervals of 100 s. The profiles of the recorded Bragg peaks were fitted with the pseudo-Voigt function using a PowderCell [25] software application (V. 2.2).

The obtained XRD spectra served to identify the crystalline components and to determine their lattice parameters using Bragg’s law. Prior to that, however, it was necessary to calculate the penetration depth of the X-ray beam. Several authors assumed the threshold sensitivity of the recorded XRD signal to be 0.1% of the incident beam and proposed a formula for the thickness of the irradiated layer that depends on the linear absorption coefficient of the material and the incidence angle [26]. Using data reported in [24], we found that the linear absorption coefficient of the WC-Co composite is 3102 cm^−1^, and the penetration depth varies from 0.679 to 9.633 µm for incidence angles in the range of 3.5 to 60°, respectively. It turns out that the maximum depth from which the XRD signal can be acquired does not exceed 10 µm for the largest incidence angles. Table 2 presents lattice parameters of the model unit cells of the crystalline sub-components of the WC-Co composite analyzed in this work.

The average grain size of the crystalline material can be determined using the equation first proposed by Scherrer [27]. This equation takes the full width at the half maximum (FWHM) of a given XRD peak and allows us to determine the size of low-angle crystallites, also called coherently diffracting domains (CDD) or low-angle mosaic blocks, in the direction normal to all parallel planes with the same (hkl) Miller indices [27]. A similar formula was proposed by Stokes and Wilson [28,29] who made use of the integral peak width instead of FWHM. However, lattice distortion also affects the width of the diffraction line. Williamson and Hall [30] assumed that the total width of the XRD peak is the sum of two components corresponding to the block size and stress level and proposed (W-H method) that the volume-weighted size of the crystallites size might be found together with the average relative lattice distortion. The main advantage of the W-H method lies in simplicity and easy interpretation of the obtained results. On the other hand, the method lacks precision in the presence of anisotropic stresses or irregular grain shapes since it can only refer to reflections of different orders originating from the same (hkl) planes. Despite those obstacles, it is still used in the studies of nanostructures, engineering ceramics, and synthetic bone tissue components, as well as semi-crystalline biological materials [19,31,32,33].

The materials under study were also analyzed in terms of the volumetric phase contributions of the crystalline components that were determined using the Rietveld refinement method [34]. Using the method of least squares, a combination of patterns of the hypothesized model structures is iteratively fitted to the recorded diffractogram resulting in fractional volumetric contents. This method replaces time-consuming analytical methods, such as the internal or external standard methods, which require measurements of stress-free reference structures with coherently diffracting domains larger than 0.1 µm, i.e., whose properties do not affect the displacement and broadening of the XRD peaks. In order to carry out the Rietveld analysis, the software application Powder Cell was used. Model crystalline structures were prepared using the Materials Project database [35].

### 2.5. Surface Roughness Measurements

Surface profile measurements were carried out using Mitutoyo Surftest SJ-210 profilometer (Kawasaki, Japan) with a scan probe with a tip of radius 2 μm. According to the norm PN-ISO 4288:2011 [36], the roughness profile should be recorded over a total length of 4 mm divided into 5 elementary sections 0.8 mm each. These measurements were repeated 5 times at various points on the surface of the drill blade. Then, from these profiles, the roughness parameters were calculated, namely: Ra, Rq, and Rz. Moreover, additional roughness profiles were taken over shorter and longer scan lengths: 0.4, 1.25, and 12.5 mm, corresponding to the following elementary sections: 0.08 mm, 0.25 mm, and 2.5 mm, respectively.

As mentioned previously, the roughness profile data placed in a descending order form a bearing area curve, which is actually a cumulative distribution function, from which the following functional parameters can be determined: R_k_—reduced core height of the roughness profile, R_pk_—reduced peak height of the roughness profile, R_vk_—reduced valley height of the roughness profile, M_r1_—profile material ratio of the peaks [%], M_r2_—profile material ratio of the valleys [%].

In addition to these two methods, the RMS method was also used [37], which yields fractal parameters from a series of the height profiles taken with varying measurement lengths. The fractal dimension for a curve lies on the spectrum between 1 and 2, with the lower limit corresponding to a straight line and the upper one to an extremely developed line with virtually infinite length. Sayles and Thomas showed [38] that the RMS roughness depends on the scan length as follows:

(2) $$R_{q}\propto l_{n}^{\frac{H}{2}}$$

where H is the Hurst exponent. On a double log plot, it can be shown from Equation (2) that for self-affine surfaces, the fractal dimension *D* can be introduced, which is related to the power exponent: $\frac{H}{2}=2-D$. When the surface obeys such a scaling law over several separate ranges with different Hurst exponents, this type of structure corresponds to multifractal or clustered structures.

## 3. Results and Discussion

### 3.1. Metallographic Analysis

**Figure 2:**
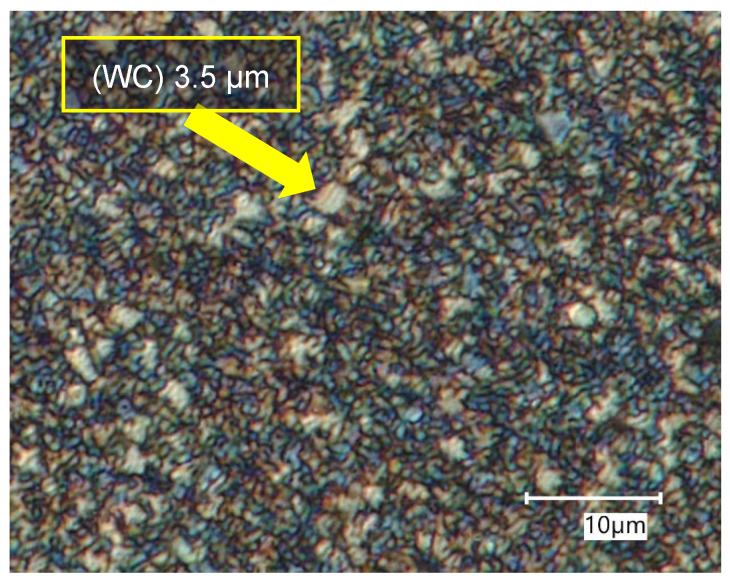
Metallographic image of the surface of the barrel drill blade.

Figure 2 shows the surface of the blade of a barrel drill that uncovers the polycrystalline structure of the sintered WC-Co composite, as seen by metallographic imaging. An image analysis reveals the predominance of grains ca. 1 µm in size in the structure of the blade of the barrel drill. Apart from that, however, larger grains reaching up to 3.5 µm in size can also be seen in the imaged area.

### 3.2. Vickers Hardness of the Cutting Blade

**Figure 3:**
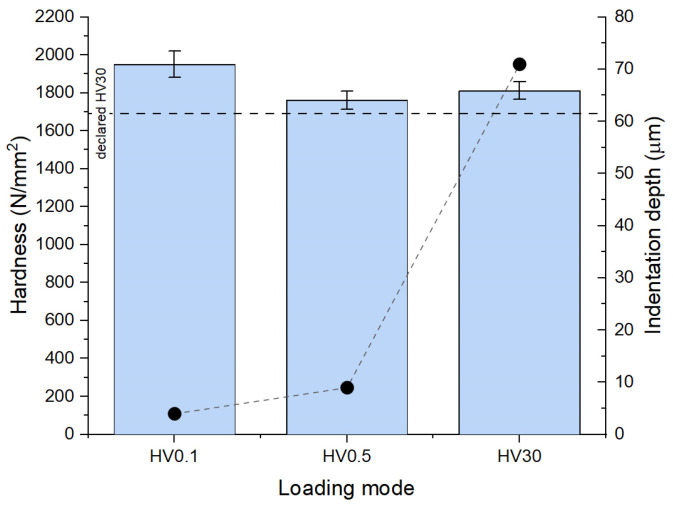
Hardness results obtained using the Vickers method with different loads (vertical bars) and estimated indentation depths of the probe (closed dots).

The obtained average hardness values of the specimens of the WC-Co composites are shown in Figure 3 (vertical bars compared with HV30 hardness declared by the manufacturer) together with indentation depths of the Vickers probe (closed dots). Obtained values depend on the indentation depth, which, in turn, varies with loading force. The following values were obtained for HV0.1, HV0.5, and HV30: 1950 ± 50, 1760 ± 50, and 1810 ± 50 N/mm^2^, respectively. Note the consistency of the hardness values measured for larger loading forces (HV0.5 and HV30) regardless of different indentation depths (9 vs. 71 μm, respectively) and the difference between these values for lower indentations depths (HV0.1 vs. HV0.5 for 4 vs. 9 μm, respectively). The observed differences likely reveal the strengthening mechanisms that affect residual layers no deeper than few micrometres. On the other hand, HV30 hardness appeared ca. 7 per cent larger than that declared by the manufacturer (1810 vs. 1690, respectively), which, in turn, might be due to the tolerance of the manufacturing process. The HV30 provided by the manufacturer shows a minimally guaranteed hardness within a series of the drill bits; however, this does not necessarily apply to the one investigated in this study. After all, for many applications, the larger the hardness of the cutting tool compared to that declared by the supplier, the better.

### 3.3. Structural Analysis by XRD

**Figure 4:**
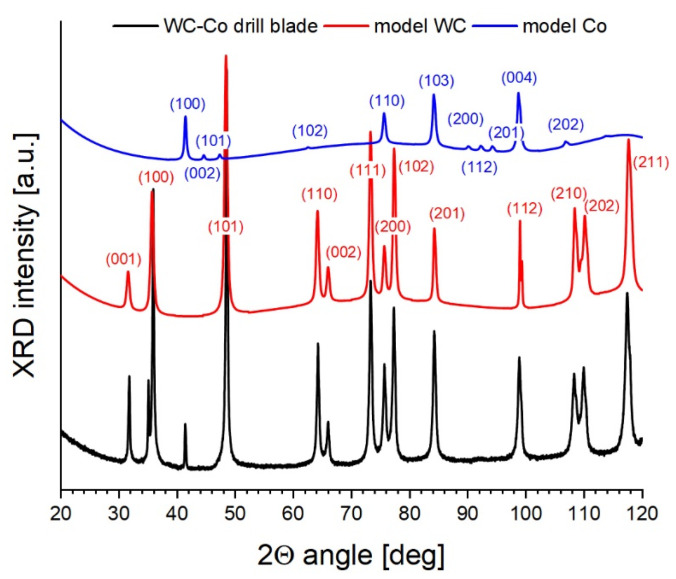
XRD diffraction patterns of the WC-Co composite in the gun barrel drill.

Figure 4 shows the recorded XRD pattern of the WC-Co composite under study (black solid line) that was then split into separate spectra of corresponding phase components (red and blue solid lines). Results of the phase analysis reported in Table 3 reveal the volumetric content of cobalt between 6.3 and 6.5%, which is very close to that declared by the manufacturers (6%). Apart from that, however, in the low-angle range of the XRD signal, significant background noise was detected, caused by small inclusions of the amorphous phase [24]. Table 3 also reports results of the iterative estimation of the lattice parameters, lattice distortions, and the size of the mosaic domains using the Williamson–Hall method.

The data in Table 3 prove that the obtained lattice parameters agree well with those taken from the Crystallography Open Database [39], corresponding to perfect, unstrained unit cells. Such a similarity is possible only in the absence of primary and secondary solid solutions within the materials under study. Both crystalline phases in the drill blade are characterized by negative lattice distortions reflecting decreased interplanar distances (dhkl) indicative of compressive (type III) intrinsic strains. This is most likely due to the manufacturing process, i.e., sintering of the carbide material under high pressure. Note also small domains found in both crystalline phases (ca. 14 nd 20 nm in WC and Co, respectively), which are 5–6 times smaller than the macro-grains seen in the metallographic image. Such a result reveals, on the one hand, the complex inner structure of single crystallites in the material being composed of sub-grains slightly tilted toward each other but tightly bound (no interfacial boundary), whereas, on the other hand, it also shows a large structure mismatch and a strongly distorted intergrain boundary between the crystallites.

### 3.4. Geometric and Functional Structure of the Surface

**Figure 5:**
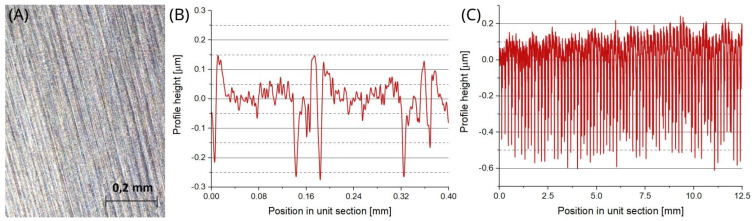
(**A**) Optical image of the surface of the blade of the barrel drill, (**B**) example roughness profile for the measurement length ln = 0.4 mm and unit section lr = 0.08 mm, (**C**) example roughness profile for the measurement length ln = 12.5 mm and unit section lr = 2.5 mm.

Figure 5A shows the optical image of the surface under study that was found to be strongly anisotropic due to grinding. Example plots of the respective roughness profiles under various magnifications are shown in Figure 5B,C.

**Figure 6:**
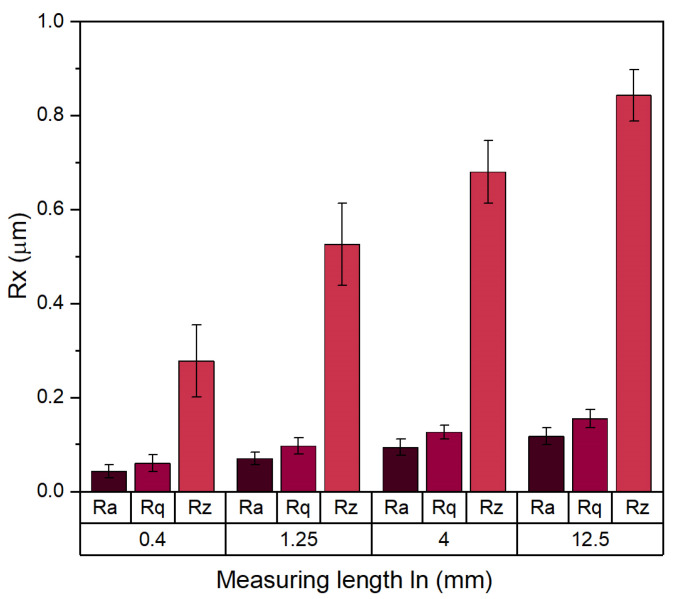
Grouped plots of surface roughness parameters for various measuring lengths: Ra—average roughness, Rq—RMS roughness, Rz—average peak-to-valley distance.

Figure 6 summarizes the roughness parameters Ra, Rq, and Rz determined for the face of the drill blade. For all these parameters, verification has been made that they come from normal distributions and that their variances were equal for all four groups assuming confidence level *p* = 0.05. On the other hand, it was also verified that all the variances in their values were statistically different. All the roughness parameters were found to be asymptotically increasing (although to a different degree) with an increasing measuring length reaching a certain limit value determined by the long-wavelength surface variations in the material. The largest roughness values were determined in the case of the Rz parameter, that is, the mean peak-to-valley distance. This suggests a large amplitude of variations in height samples especially on the waviness (long-wavelength) level.

**Figure 7:**
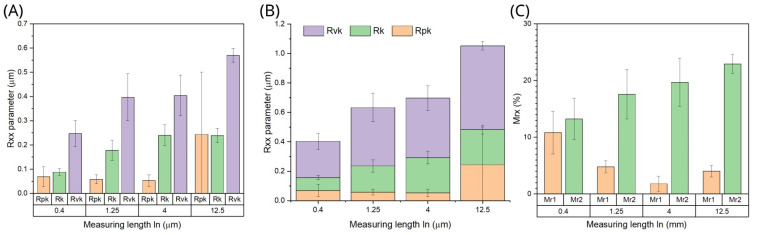
Grouped and stacked plots of functional roughness parameters for various measuring lengths: R_k_—reduced core height of the roughness profile, R_pk_—reduced peak height of the roughness profile, R_vk_—reduced valley height of the roughness profile, M_r1_—profile material ratio of the peaks, M_r2_—profile material ratio of the valleys. (**A**) grouped plot of the Rxx parameters vs. measuring length (**B**) same data plotted in a stacked form (**C**) grouped plot of Mrx parameters vs measuring length.

On the other hand, the roughness profiles also served to compute the BAC curves from which functional parameters have been derived. Obtained results are summarized in Figure 7. Note the superior properties of Rpk in terms of accumulation of the lubricant.

A graphical summary of the reduced heights of the functional layers of the drill blade shown in Figure 7A,B exhibits significantly deeper valleys than the highest peaks. Remember that Rpk characterizes the outermost parts of the surface that become worn first during contact work, which can be used as a measure of abrasive wear resistance in the lapping phase. On the other hand, Rvk defines the ability of the surface to accumulate the lubricant, while Rk is the height of the core layer responsible for most of the bearing load. That said, for the measurement length 0.4 mm, the Rpk thickness is only 28% that of the Rvk, and for the increasing measurement lengths: 1.25, 4.0, and 12.5 mm, this ratio achieves 15, 13, and 43%, respectively. This proves the ability of the surface of the blade to retain lubricant fluid, hence, reducing the wear of touching surfaces which is highly beneficial for tool materials.

In turn, Figure 7C provides a comparison of the material ratios of the peaks M_r1_ and valleys M_r2_ (actually: complemented to 100%) in the roughness profiles. In this figure, the gap can be found between the ratio of the highest peaks Mr1 and the deepest valleys (100—Mr2) in the outer surface layer that gradually increases with increasing measuring length, i.e., it is the largest for the long-wave height variability components. In order to estimate the total volume of each layer, the material ratios and corresponding thicknesses of the functional layers in the roughness profiles should be analyzed together. It turns out that the volumetric contributions of the peaks and valleys in roughness profiles have similar values regardless of the measurement length.

**Figure 8:**
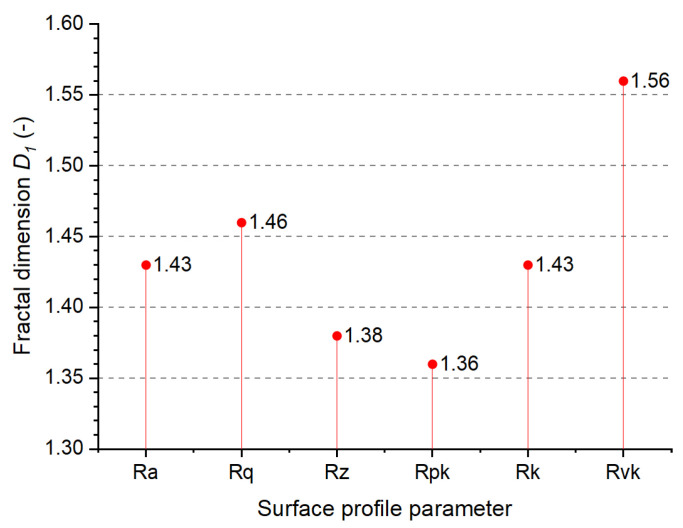
Plot of fractal dimensions determined from the allometric dependencies of the roughness parameters on the measuring length.

In order to analyze the geometric structure of the surfaces under study in terms of multi-scale fractal parameters, the abovementioned RMS methodology was applied. Figure 8 shows the results of the performed analysis indicating the monofractal character of the surface layer of the drill blade and its self-similarity within the entire range of the measuring lengths, i.e., from 0.4 to 12.5 mm. The same RMS method was also applied to the analysis of the functional parameters Rpk, Rk, and Rvk, which is also shown in Figure 8. The fractal dimensions estimated from these parameters appear nearly similar to those estimated from the roughness parameters that confirm the monofractal structures of the functional surface layers of the sintered WC-Co composite. After all, the mean fractal dimension is found to be 1.44 ± 0.07, specific to moderately developed surfaces.

**Figure 9:**
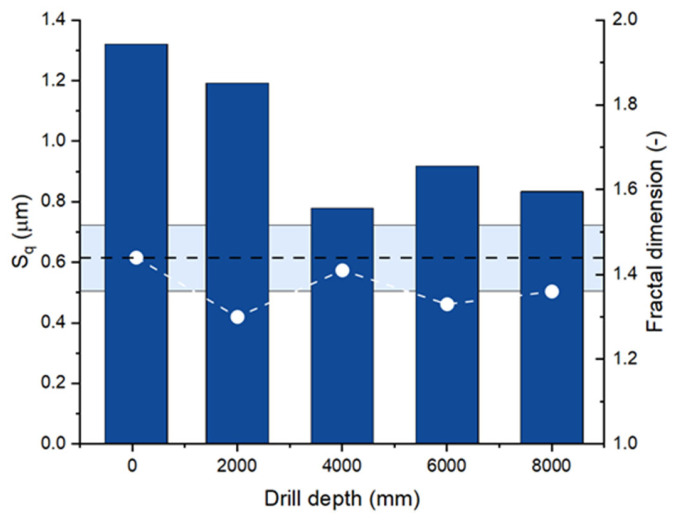
Plot of the surface roughness Rq of the bores drilled in the C45 steel block vs. the drilling depth (blue vertical bars) and the fractal dimensions of these surface profiles (white dots). In addition, the average fractal dimension of the drill blade (1.44 ± 0.07) is shown (dashed line with uncertainty blueish bar).

Finally, Figure 9 shows the changes in the Rq roughness and fractal dimension of the surface of the drill bore made in C45 steel using the barrel drill under study. The total drilling depth was 8000 mm, and the surface roughness was measured every 2000 mm. It turns out that the roughness gradually decreased from 1.3 to 0.9 μm, but the fractal dimension oscillated between 1.25 and 1.45, decaying to ca. 1.35 in the end of the drilling process. For comparison, also the initial fractal dimension of the barrel drill is shown (1.44 ± 0.07, blueish horizontal bar), which agrees well with those of the machined steel. Observed self-consistency of the fractal dimensions of the two materials in intense contact proves that any tool leaves its characteristic fingerprint on the residual surface layer of the material and vice versa.

## 4. Conclusions

The outer surface of the drill blade made of the WC-Co composite appears monofractal in terms of various roughness and functional parameters. The consistency of the fractal dimensions estimated is due to the finer finishing that spanned over several orders of the wavelength. The application of the RMS method made it possible to investigate the scalability of the topography of the tested surfaces and to establish the dependence of the roughness and load-bearing parameters on the measurement length. It was also proven that materials in contact affect each other in terms of obtained fractal characteristics such that during the prolonged machining of the C45 steel substrate, the fractal dimensions of the drill blade and the bore become similar.

## Figures and Tables

**Table 1:** Declared specification of the tool material of the gun barrel drill under study.

Parameter	EB80 GÜHRING
Material	Sintered WC
Type	K15
Chemical composition (% vol)	WC 94/Co 6
Density	14,850 kg/m^3^
Granularity	1.0 μm
Hardness (HV30)	1690

**Table 2:** Prototype crystal lattice data of the structures in the WC-Co composite.

Lattice Parameters [Å]	Spatial Group No. (Symmetry)	Element	Position of the Atom in the Unit Cell			Unit Cell
			x	y	z	
**WC**						
a_0_ = 2.9065 c_0_ = 2.8366 γ_0_ = 120°	187 (P-6m2)	W	0	0	0	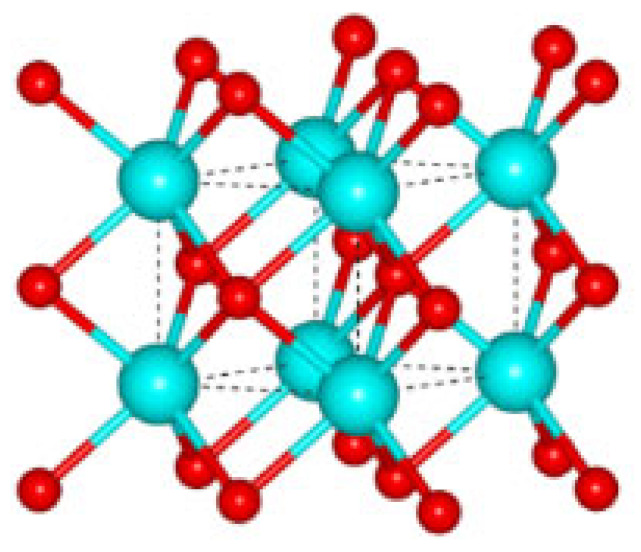
		C	1/3	2/3	1/2	
**Co**						
a_0_ = 2.5071 c_0_ = 4.0686 γ_0_ = 120°	194 (P6_3_/mmc)	Co	1/3	2/3	1/4	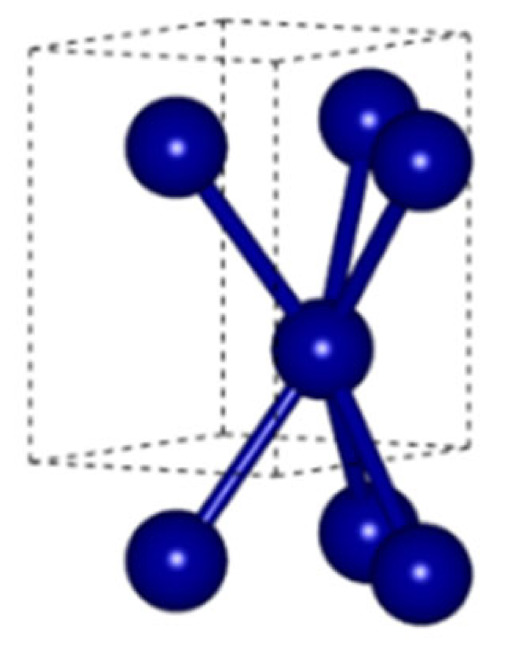

**Table 3:** Phase composition and lattice parameters of the crystalline structures in WC-Co composites.

Crystalline Phase	Initial Lattice Parameters [Å]	Final Lattice Parameters [Å]	Volumetric Phase Content [%]	Domain Size [Å]	Lattice Distortion [%]
WC	a_0_ = 2.9065 c_0_ = 2.8366 γ_0_ = 120°	a_0_ = 2.9023 c_0_ = 2.8292 γ_0_ = 120°	93.53	141.26	−0.065
Co	a_0_ = 2.5071 c_0_ = 4.0686 γ_0_ = 120°	a_0_ = 2.5098 c_0_ = 4.0550 γ_0_ = 120°	6.47	199.09	−0.04

## Data Availability

The original contributions presented in this study are included in the article. Further inquiries can be directed to the corresponding author.
